# New Appliances and Things Medical

**Published:** 1895-08-31

**Authors:** 


### NEW APPLIANCES AND THINGS MEDICAL.

[We shall be glad to receive, at our Office, 428, Strand, London, W.C., from the manufacturers, specimens of all new preparations and appliances which may be brought out from time to time.]

### PATENT BARLEY.—PATENT GROATS.—WAVERLEY WAFERS.

(Keen, Robinson, and Co., Limited, London).

The patent barley, which has already received 41 prize medals and three Royal warrants, is a beautiful preparation and absolutely pure. As an infant's food it is invaluable. Barley water should first be made by boiling a dessert spoonful of patent barley with a pint of water. After half an hour's boiling it should be allowed to cool, the skin should be removed and diluted with various proportions of milk, according to the age and digestive powers of the infant. The patent barley may further subserve several other domestic purposes. From it excellent custard puddings and blancmanges may be made, and for the brewing of invalid and summer beverages it has no rival. The patent groats which are prepared by the same celebrated firm serve as a valuable food for nursing mothers and young children. It may be offered in the form of gruel, posset, or as a substitute for porridge, possessing, like the latter, the valuable property of stimulating the bowel and relieving constipation. The Waverley wafers, also prepared by Messrs. Keen and Robinson, have valuable nutritive properties, and by following the recipes supplied with the wafers the most inexperienced person can turn out most delicious and novel varieties of scone, pudding, or biscuit.

